# A retrospective, multicenter study on the management of macular holes without residual internal limiting membrane: the refractory macular hole (ReMaHo) study

**DOI:** 10.1007/s00417-022-05739-x

**Published:** 2022-07-06

**Authors:** Umberto Lorenzi, Joel Mehech, Tommaso Caporossi, Mario R. Romano, Rocco De Fazio, Eric Parrat, Frédéric Matonti, Paolo Mora, on behalf of the ReMaHo Study Group, Giancarlo Sborgia, Matteo Forlini, Luca Ventre, Vincent Soler, Magali Sampo, Tito Fiore, Koen Van Overdam, Sébastien Guigou, Hervé Rouhette, Emilio Rapizzi, Eric Denion, Olivier Rebollo, Franck Meyer, Joel Uzzan, Marco Mafrici, Daniela Bacherini, Stefania Favilla, Guido Ricciotti, Salvatore A. Tedesco, Stefano Gandolfi, Marc Muraine

**Affiliations:** 1 Ophthalmology Unit, Centre Hospitalier Universitaire Charles-Nicolle, Rouen, France; 2 P 1.5 Group, 80, allée des Ormes, 06250 Mougins, France; 3 Ophthalmology Unit, Fondazione Policlinico Universitario A. Gemelli IRCCS, Rome, Italy; 4 Catholic University “Sacro Cuore”, Rome, Italy; 5 Department of Biomedical Science, Humanitas University, Pieve Emanuele, Milan, Italy; 6 Ophthalmology Unit, Ospedale Santa Maria della Scaletta, Imola, Italy; 7 Clinique Des Eaux Claires, Baie-Mahault, Guadeloupe France; 8 Centre Monticelli Paradis, Marseille, France; 9 Institut de Neuroscience de La Timone, Aix-Marseille University, Marseille, France; 10 Clinique Juge, Groupe Almaviva Santé, Marseille, France; 11 Ophthalmology Unit, University Hospital of Parma, 43126 Parma, PR Italy

**Keywords:** Full thickness macular hole, No internal limiting membrane, Reconstructive surgery, Prognostic variables

## Abstract

**Purpose:**

To evaluate the surgical management, outcomes and prognostic factors of full thickness macular holes without residual internal limiting membrane (NO-ILM FTMHs).

**Methods:**

We performed a multicenter, retrospective study of 116 NO-ILM FTMHs. Human amniotic membrane (hAM) plug, autologous ILM free flap transplantation (AILMT), and autologous retinal graft transplantation (ART) were performed in 58, 48, and 10 patients, respectively. Data were collected before and up to 12 months after surgery. The primary outcomes were hole closure and final best-corrected visual acuity (BCVA).

**Results:**

The final BCVA (0.78 ± 0.51 logMAR) was significantly better than and correlated with the initial BCVA (p < 0.0001 and p = 0.004, respectively). Hole closure was achieved in 92% of eyes. The minimum FTMH diameter was wider and final BCVA was lower in the ART group than in the other groups (p < 0.003 and p < 0.001, respectively). FTMHs with diameter > 680 μm had a higher closure rate with hAM than with AILMT (p = 0.02).

**Conclusions:**

AILMT and hAM were the most frequently performed surgeries with both high closure rate and significant functional improvement. Preoperative BCVA was correlated with final BCVA. The minimum FTMH diameter may guide the treatment choice.


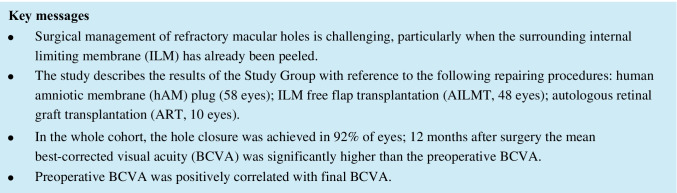


## Introduction

Full thickness macular hole (FTMH) is a retinal defect that involves the fovea and causes significant visual deterioration. FTMH may be related to trauma, high myopia, or posterior ocular surgery. However, it is idiopathic in most patients. Pars plana vitrectomy (PPV) with internal limiting membrane (ILM) peeling, gas tamponade, and prone positioning leads to anatomical closure in approximately 95% of patients [1].

FTMH is classified as refractory if the hole is not closed by surgery or re-opens after surgical closure [2]. Primary repair of FTMH has a high success rate and refractory FTMH occurs in a small number of patients. Anatomical and functional recovery is difficult to achieve in patients with refractory FTMH. Refractory FTMHs are associated with chronic and large holes, high myopia, non-idiopathic FTMHs, Afro-Caribbean ethnicity, short duration of intraocular gas tamponade, incomplete vitrectomy, and failure to comply with postoperative head positioning [2–8]. Repeat PPV with enlargement of previous ILM rhexis, where possible, is a commonly used second-line treatment [9, 10].

During the past decade, various reconstructive techniques have been used to treat refractory FTMHs. The choice of reconstructive techniques depends on equipment availability, personal experience, and patient characteristics. Autologous ILM free flap transplantation (AILMT) can be performed in the absence of residual ILM around the macula; it involves microstructural reconstruction in the FTMH area [11–13]. Human amniotic membrane (hAM) grafts are also frequently applied for the treatment of refractory FTMHs [14]. In addition, autologous retinal graft transplantation (ART) has been used for patients with large and chronic FTMHs, concomitant retinal detachment, or previous extensive ILM peeling [15–17]. Other procedures proposed for refractory FTMHs include autologous thrombocyte serum concentrate administration, anterior/posterior lens capsule transplantation, strategies to make the retina more elastic and/or mobile on the pigment epithelium layer (e.g., macular hole hydrodissection) [18–20].

This study was performed to assess the practices of vitreoretinal surgeons with similar age, training centers, and experience regarding the management of complex FTMH cases in which macular ILM peeling or re-peeling has previously been performed. The results of the three most commonly used reconstructive techniques were evaluated in terms of anatomical and functional outcomes. In addition, possible associations of pre- and intraoperative variables with outcomes were assessed.

## Methods

**Fig. 1:**
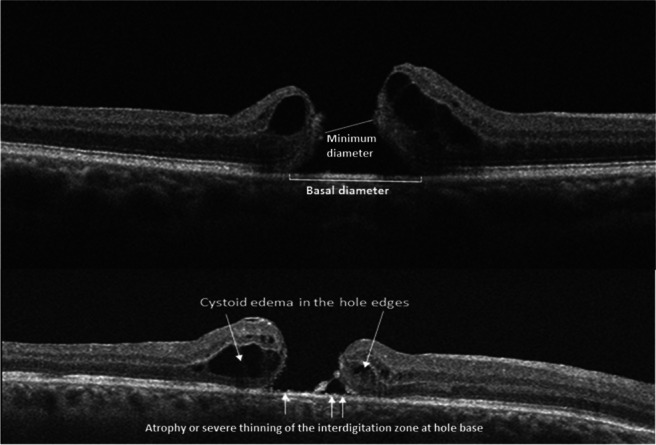
Reference optical coherence tomography images to standardize measurements and surveys on the de-identified data records

We conducted a multicenter, retrospective case series involving surgeons working in the South-Central Europe and French West Indies areas. Surgeons were requested to report cases of FTMH without residual ILM within approximately a two-disc diameter around the hole edge because of ILM peeling performed during prior vitreoretinal interventions (i.e., NO-ILM FTMHs). Eligible patients had to be followed up postoperatively for ≥ 12 months and suitable macular imaging obtained preoperatively and 3, 6, and 12 (± 1) months postoperatively. We excluded patients with age < 18 years; postoperative complications preventing fundus imaging and/or requiring further vitreoretinal surgery (e.g., hemorrhage, endophthalmitis, retinal detachment) except silicone oil removal; postoperative graft dislodgement. Surgeons were provided with a common Excel sheet for data entry and reference optical coherence tomography images to standardize measurements and surveys on the de-identified data records (Fig. 1). Surgeries were performed between January 1, 2017, and June 30, 2020. The study adhered to the Declaration of Helsinki and was approved by the Institutional Ethics Committee (protocol number E2021-28) of the coordinating center (University Hospital of Rouen, France).

### Surgical procedures

Study surgery was considered “first attempt” if it aimed to close a naïf NO-ILM FTMH (i.e., the ILM had been peeled during previous interventions to treat different posterior segment diseases); or “true refractory” if it aimed to manage holes that were either not closed or reopened after repairing surgery. The patients underwent transconjunctival PPV under general anesthesia or retrobulbar/peribulbar anesthesia.

- *AILMT*: The blue dye (brilliant blue G alone or combined with trypan blue) was applied to the withdrawing area for approximately 30 s; an ILM patch was harvested, preferably from the edge of the previously peeled area, to fit the FTMH width. The free flap was dragged over (cover technique) or pushed below (fill-in technique) the hole edges, possibly under dense matrix to stabilize the plug [11, 21].
- *ART:* ART was performed using a unimanual or bimanual technique and a single- or dual-fiber endoillumination system. A 2 disc-diameter area of the retina was circumscribed by endolaser barricade; endodiathermy was applied to blood vessels at the edges of the so delimitated area. A localized retinal detachment by subretinal fluid injection could be induced before harvesting the graft by end-grasping forceps and curved or vertical scissors. Eighty percent of grafts were taken from the superior mid-periphery except in two patients with associated rhegmatogenous retinal detachment, in whom the retinal patch was obtained directly from the edge of a leaking tear. The retinal free flap was transferred to the recipient site to cover or fill the FTMH [15, 22].
- *hAM plug:* The plug was shaped from the donor tissue and inserted into the vitreous cavity through a valveless trocar or by temporary removal of the trocar. The hAM was positioned inside the hole, preferably with the basal membrane facing upward and the chorion oriented toward the retinal pigment epithelium (RPE).

The rate of “covered” or “filled” cases, substances used to stabilize the plug, final tamponade, and duration of face positioning were recorded for all techniques.

### Examinations

The primary outcomes were anatomical NO-ILM FTMH closure and best-corrected visual acuity (BCVA) at the end of the 12-month follow-up period. BCVA was measured in logMAR units using the Early Treatment of Diabetic Retinopathy Study (ETDRS) chart. The secondary outcomes analyzed BCVA trend, and the possible statistical correlation between primary outcomes and: a) imaging findings (e.g., minimum and basal diameter of NO-ILM FTMH; b) some preoperative and intraoperative variables, such as demographics, axial length, hole type (i.e., first attempt or true refractory), probable causative pathology, number of previous repairs (0 for first attempt surgeries), medium under which the plug was positioned, final tamponade, duration of postoperative head positioning.

On the images surgeons recorded preoperative diameters (μm), presence/absence of cystoid edema or of detachment in the hole edges, atrophy or severe thinning of the interdigitation zone (IZ) at hole base; postoperative FTMH closure (i.e., type 1 of Kang’s classification) [23], and restoration of the external limiting membrane (ELM) (continuous, partial, or absent). Cases with uncertain classification were independently reviewed by two authors (UL and PM).

### Statistical analysis

Means and standard deviations were reported for continuous variables with a normal distribution, whereas medians with interquartile ranges were reported for continuous variables with a non-normal distribution. Linear regression models were used to test correlations. All the collected variables were assessed for potential correlations with the primary outcomes and included in the multivariate analysis. It has to be noted that, by default, most of the regression models require that all the database entries are fulfilled to work; that is, they exclude the cases with missing covariates. Between- and within-group analyses were performed using repeated-measures analysis of variance. Post hoc contrast analysis was performed using Fisher’s least significant difference test (95% family-wise confidence level). Analyses were performed using linear regression models in the R Cran environment (https://cran.r-project.org/ ver. 3.4.0) and p-values < 0.05 were considered statistically significant [24]. 

## Results

The study included PPV procedures (60 and 56 using 23-G and 25-G calipers, respectively) performed by 21 surgeons on 116 eyes of 116 patients (70 women and 46 men; mean age: 65 years; age range: 18–86 years). Simultaneous phacoemulsification was performed in the 12 eyes which still preserved the natural lens at the time of the study surgery. Preoperatively, the mean BCVA was 1.14 (± 0.48) logMAR, median axial length was 24.35 mm (interquartile range: 23.11–30.01 mm), and mean minimum and basal FTMH diameters were 630 (± 286) and 975 (± 421) μm, respectively. First attempt and true refractory procedures were performed in 19 (16%) and 97 (84%) eyes, respectively. The true refractory procedures included 2 recurrent (i.e., re-opened) cases; 78, 18, and 1 eyes had undergone one, two, and three previous surgeries. Most of these pre-study repairing attempts (83%) involved ILM peeling (with or without the “inverted flap” maneuver). The holes were idiopathic (89 eyes) or secondary to previous retinal detachment (20 eyes), non-penetrating ocular trauma (4 eyes), macular neovascular membrane (2 eyes), or epiretinal membrane surgery (1 eye). Preoperative imaging assessing detected cystoid edema in the hole edges in 60 eyes (52%), IZ atrophy at the FTMH base in 43 eyes (37%). Postoperatively, cystoid macular edema was present at two or more follow-up time points in 5 hole-closed eyes despite topical and local treatments, whereas 1 eye developed steroid-dependent panuveitis.

The final postoperative BCVA was 0.78 (± 0.51) logMAR, which was significantly improved from the baseline value (p < 0.0001). FTMH was successfully closed in 107 eyes (92%). The association of final BCVA and FTMH closure with sex (internal control for fitness), surgeon, surgical technique, age at surgery, clinical characteristics, causative pathology, number of previous surgical repair attempts, duration of postoperative head positioning, tamponade, and preoperative characteristics (i.e., preoperative BCVA, axial length, and FTMH diameters on optical coherence tomography) are tested and shown in Tables 1 and 2. The final BCVA was significantly, positively correlated with initial BCVA (p = 0.004). However, FTMH closure was not correlated with any of the aforementioned variables.

**Table 1:** Best-corrected visual acuity (BCVA) at the end of the follow-up: full regression model output

Outcome: BCVA final	Coefficients:	Estimate	S.E	t value	p-value
(Intercept)	-	1.29E + 03	5.83E + 02	2.209	0.032*
Surgical Technique (baseline AILMT)	hAM	3.95E + 01	1.56E + 02	0.253	0.801
	ART	4.27E + 02	2.68E + 02	1.596	0.117
Surgeon (baseline S1)	S2	-1.04E + 02	3.01E + 02	-0.344	0.732
	S3	7.25E + 02	4.48E + 02	1.621	0.111
	S4	-7.63E + 02	4.23E + 02	-1.803	0.077
	S5	-3.11E + 02	3.11E + 02	-1.001	0.322
	S6	-7.22E + 01	4.28E + 02	-0.169	0.867
	S7	-4.68E + 02	2.91E + 02	-1.611	0.113
	S8	-2.34E + 02	4.19E + 02	-0.559	0.579
	S9	-6.73E + 01	3.57E + 02	-0.189	0.851
	S10	-1.67E + 02	3.22E + 02	-0.520	0.605
	S11	-4.70E + 02	3.16E + 02	-1.490	0.143
	S12	-1.71E + 02	4.18E + 02	-0.408	0.685
	S13	-4.27E + 02	3.34E + 02	-1.276	0.208
	S14	-3.01E + 02	3.91E + 02	-0.769	0.446
	S15	-2.65E + 02	3.19E + 02	-0.831	0.410
Sex (baseline F)	M	-6.89E + 01	6.86E + 01	-1.004	0.320
Age	-	-3.17E + 00	3.20E + 00	-0.989	0.327
BCVA preoperative	-	3.04E + 02	1.01E + 02	3.010	0.004**
Axial length	-	-1.11E + 01	1.14E + 01	-0.972	0.336
FTMH Diameter (min)	-	5.89E-02	1.40E-01	0.422	0.675
Cystoid Edema	-	-5.53E + 01	7.96E + 01	-0.695	0.491
EPR Atrophy	-	1.85E + 01	8.07E + 01	0.229	0.820
Macular detachment	-	2.60E + 02	1.60E + 02	1.625	0.111
Causative pathology (baseline Idiopathic)	Retinal detachment	-2.11E + 01	1.69E + 02	-0.125	0.901
	Traumatic	-8.20E + 01	3.80E + 02	-0.216	0.830
n° previous surgery	0–3	2.56E + 00	8.20E + 01	0.031	0.975
TAMP (baseline air)	Gas	-7.41E + 01	1.06E + 02	-0.699	0.488
	PDMS	-2.94E + 02	2.23E + 02	-1.318	0.194
Face down (days)	-	-2.84E + 01	2.91E + 01	-0.976	0.334

The baseline category represents the reference against which the model compares all the other categories

S.E.: standard error; AILMT: autologous internal limiting membrane free flap transplantation group; hAM: human amniotic membrane group; ART: autologous retinal graft transplantation group. FTMH: full thickness macular hole; TAMP: type of tamponade (air/Gas/PDMS)

**Table 2:** Open/Close status of the full thickness macular hole (FTMH) at the end of the follow-up: full regression model output

Outcome: Open/Close	Coefficients:	Estimate	S.E	t value	p-value
(Intercept)	-	0.609	0.705	0.865	0.392
Surgical Technique (baseline AILMT)	hAM	0.031	0.184	0.168	0.867
	ART	0.125	0.314	0.396	0.694
Surgeon (baseline S1)	S2	0.120	0.355	0.339	0.736
	S3	-0.050	0.533	-0.094	0.926
	S4	0.037	0.498	0.075	0.941
	S5	0.280	0.372	0.753	0.455
	S6	0.020	0.504	0.040	0.968
	S7	0.177	0.342	0.518	0.607
	S8	-0.097	0.494	-0.197	0.845
	S9	0.323	0.420	0.769	0.446
	S10	0.333	0.380	0.875	0.386
	S11	0.142	0.456	0.311	0.757
	S12	-0.209	0.491	-0.425	0.673
	S13	0.062	0.397	0.156	0.877
	S14	-0.212	0.460	-0.461	0.647
	S15	-0.101	0.376	-0.267	0.790
Sex (baseline F)	M	-0.102	0.084	-1.212	0.232
Age	-	-0.001	0.004	-0.394	0.695
BCVA preoperative	-	-0.052	0.120	-0.436	0.665
Axial length	-	-0.014	0.016	-0.871	0.388
FTMH Diameter (min)	-	0.000	0.000	1.200	0.236
Cystoid Edema	-	-0.035	0.093	-0.372	0.711
EPR Atrophy	-	0.017	0.098	0.172	0.864
Macular detachment	-	0.126	0.191	0.656	0.515
Causative pathology (baseline Idiopathic)	Ret. detachment	0.049	0.202	0.240	0.812
	Traumatic	0.113	0.450	0.252	0.802
n° previous surgery	0–3	0.021	0.097	0.221	0.826
TAMP (baseline Air)	Gas	-0.086	0.125	-0.693	0.492
	PDMS	-0.225	0.263	-0.858	0.395
Face down (days)	-	-0.034	0.034	-1.001	0.322

The baseline category represents the reference against which the model compares all the other categories

S.E.: standard error; AILMT: autologous internal limiting membrane free flap transplantation group; hAM: human amniotic membrane group; ART: autologous retinal graft transplantation group. FTMH: full thickness macular hole; TAMP: type of tamponade (air/Gas/PDMS)

**Fig. 2:**
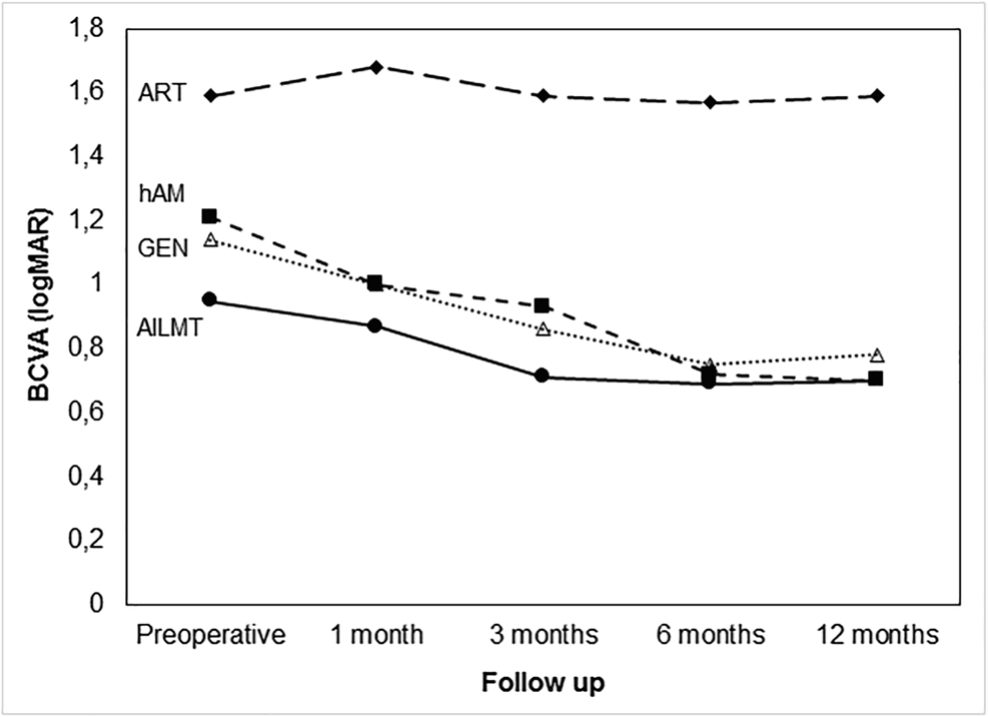
Visual acuity variations in the whole cohort and in each surgical subgroup during the follow-up*.* BCVA: best-corrected visual acuity (in logMAR units); GEN: the whole cohort; AILMT: autologous internal limiting membrane free flap transplantation group: ART: autologous retinal graft transplantation group; hAM: human amniotic membrane group

Fifty-eight, 48, and 10 eyes underwent hAM, AILMT, and ART. Table 3 summarizes the main characteristics of each surgical group. The three groups differed in terms of sample size, which was adjusted during statistical analysis. In addition, statistical differences concerned: a) hole minimum diameter, wider in the ART group (p < 0.003); b) initial BCVA (any subgroup); c) final BCVA, lower in the ART group (p < 0.001). Figure 2 shows the BCVA variation in the whole cohort and each subgroup. In the ART group, BCVA did not significantly change during follow-up. However, in the hAM and AILMT groups, BCVA significantly improved after the first and third postoperative months, respectively; this improvement continued until the sixth month then stabilized. Analysis of the other parameters (data not included in the Tables) showed that in the AILMT group, absence of IZ anomalies at the hole base was correlated with higher final BCVA (p = 0.04).

**Table 3:** Characteristics of eyes in the three treatment groups

Surgical Technique	AILMT *	hAM †	ART ‡
Treated eyes	48	58	10
Age at study surgery (yr.)	64 ± 12	66 ± 12	68 ± 11
Axial length (mm)	24.81 ± 3.08	26.81 ± 3.69	26.30 ± 2.84
BCVA preop. (logMAR)	0.95 ± 0.38†‡	1.21 ± 0.45*‡	1.59 ± 0.66*†
FTMH min. diameter (μm)	551 ± 215	684 ± 273	881 ± 514*†
FTMH bas. diameter (μm)	801 ± 413	1058 ± 386	1101 ± 469
Previous surgeries (mean)	1.0	1.1	0.8
Tamponade (Oil–Gas–Air)	2—46—0	3—40—15	7 – 3—0
Face positioning (days)	5.2	4.6	4.9
BCVA final (logMAR)	0.70 ± 0.47	0.70 ± 0.34	1.59 ± 0.86*†
FTMH closed	43 (90%)	54 (93%)	10 (100%)

Each surgical group has an identifying symbol: * for the autologous internal limiting membrane free flap transplantation (AILMT) group; † for the human amniotic membrane (hAM) group; ‡ for the autologous retinal graft transplantation (ART) group. The presence of one or two symbols next to a value in the three-column format marks the significant difference with respect to the group/s identified by the symbol/s

BCVA: best-corrected visual acuity; FTMH: full thickness macular hole; min. diameter: minimum diameter; bas. diameter: basal diameter

For the AILMT and hAM groups, we arbitrarily set at 680 μm a cutoff of hole width to evaluate the closure rate above and below this threshold (Table 4); the ART group was not considered because of the preoperative minimum diameter significantly larger. Concerning holes with minimum diameter ≤ 680 μm, the 100% closure rate achieved with AILMT did not significantly differ from that of the hAM group (90%). Among cases with diameter > 680 μm, the closure rate was significantly higher in the hAM group than in the AILMT group (p = 0.02). Based on visual improvement, AILMT and hAM showed comparable results, regardless of the diameter threshold.

**Table 4:** Rate of closure of the NO-ILM FTMHs referred to an arbitrary set minimum diameter threshold

	Minimum diameter ≤ 680 µm		Minimum diameter > 680 µm	
	OPEN *(%)*	CLOSED *(%)*	OPEN *(%)*	CLOSED *(%)*
AILMT	0	100	35.7	64.3
hAM	9.7	90.3	4.5	95.5
p-value (AILMT vs hAM)	0.1		0.02*	

NO-ILM FTMHs: full thickness macular holes without residual internal limiting membrane; AILMT: autologous internal limiting membrane free flap transplantation group; hAM: human amniotic membrane group

The fill-in technique was the most frequently used method to secure the graft position (92%, 98%, and 70% in the AILMT, hAM, and ART groups). In the hAM group, sulfur hexafluoride was the most commonly used gas for tamponade. After 12 months, in none of the closed NO-ILM FTMHs the ELM line was clearly detectable. Concerning AILMT, in 90% of the procedures the patch was positioned under dense medium (60% perfluorocarbon liquid and 40% viscoelastic matrix); in all surgeries except for one, was used gas tamponade (sulfur hexafluoride in 23 eyes and perfluoropropane C3F8 in 19 eyes). After 12 months, the ELM line was detectable and continuous in 9 of the closed cases (21%). Concerning ART, in 70% of the procedures the plug was dragged under dense medium (repartition as above); low-density silicone oil tamponade was used in 70% of the cases and gas in the remaining 3 eyes. After 12 months, the ELM line was detectable and continuous in 1 eye.

## Discussion

The present study aimed to evaluate the practices of surgeons regarding the treatment of NO-ILM FTMH cases. We evaluated the possible correlations of primary outcomes with preoperative and intraoperative variables.

Most NO-ILM FTMH cases were originally idiopathic (77%) and true refractory (84%); they had undergone a median of one previous surgery. In 50%, 41%, and 9% of the cases, hAM plug, AILMT, and ART were performed, respectively. These results suggest that the reported trend to address chiefly very large, chronic or unusual FTMHs by ART was likely followed by the authors [16, 17]. The closure rate was high for all procedures. Among all groups, the final BCVA significantly improved (i.e., mean gain of > 3 lines on the ETDRS chart, compared to baseline). Preoperative BCVA was the only parameter that exhibited a correlation with the final visual acuity. Considering the subgroup analyses, the final BCVA was significantly lower in the ART group than in the other groups [2, 25]. The smaller number of cases, larger minimum diameter, and significantly lower initial BCVA in the ART group (Table 3) does not allow us to further comment on the results of this technique.

A clearly detectable ELM line at 12 months was most commonly observed in the AILMT group (21%). In addition, AILMT showed significant positive correlations between final BCVA and the absence of preoperative IZ atrophy at the hole base (p = 0.04). Integrity of the IZ/RPE complex at the FTMH base may positively affect subsequent reconstruction processes and the final BCVA, particularly in cases of AILMT. In the hAM group, BCVA significantly improved after the first postoperative month, while in the AILMT group, BCVA improved after the third postoperative month. After 6 months postoperatively, the BCVA did not significantly change. The FTMH closure rate and final BCVA in the AILMT and hAM groups in the present study are similar to the findings in previous reports [12, 14]. Because the mean preoperative FTMH diameters were significantly larger in the present study than the conventional threshold to define “large” FTMHs, we compared the closure rates of cases with minimum diameters larger and smaller than an increased threshold (680 μm). We only compared the AILMT and hAM groups because the baseline minimum diameter was significantly larger in the ART group than in the other groups. The closure rate for FTMHs with diameter ≤ 680 μm was similar for AILMT and hAM techniques; however, for FTMHs with diameter > 680 μm, hAM had a higher closure rate than did AILMT (p = 0.02). ILM is extremely delicate and often harvested in small pieces, which complicates the repair of wide NO-ILM FTMHs. In contrast, hAM is performed using wide donor plugs, making it suitable for large (more than for small) holes. The preoperative FTMH size did not affect the postoperative gain in visual acuity. However, the visual improvement was greater for small holes treated with AILMT and for large holes (i.e., > 680 μm) treated with hAM.

The following study limitations should be considered. The retrospective study design may have introduced bias in terms of participant enrollment. In addition, some ancillary preoperative observations were not available for all eyes, which prevented a comprehensive knowledge of the medical and surgical history of patients with refractory FTMHs. While the study eyes were all pseudophakic, the presence of varying degrees of posterior capsule opacification or the occurrence of laser capsulotomy during the follow up may have affected the final BCVA. The number of patients varied among surgical subgroups. In particular, the ART subgroup had a small number of patients. Although adjustments were performed during statistical analysis to control for variation in sample size, correlation analysis could have been affected. In addition, the study design did not allow assessing intraoperative complications because of the exclusion of cases with impaired fundus imaging or requiring additional surgical management (e.g., patients with hemorrhage, endophthalmitis, and retinal detachment).

In conclusion, we analyzed 116 cases of reconstructive surgery for NO-ILM FTMH repair, which were not eligible for ILM peeling. AILMT and hAM were the most frequently performed procedures and allowed both anatomical and functional significant improvements. The preoperative visual acuity was significantly correlated with the final BCVA. The minimum FTMH diameter may be used to guide the choice of appropriate treatment, but it should not be neglected that, although anatomical closure can be achieved also in very large holes, visual recovery is very limited in these cases.

## Appendix

Other members of the ReMaHo study group:

Giancarlo, Sborgia, MD, PhD^9^; Matteo, Forlini, MD^10^; Luca, Ventre, MD^11^; Vincent, Soler, MD, PhD, FEBO^2,12^; Magali, Sampo, MD^2,13^; Tito, Fiore, MD^14^; Koen, Van Overdam, MD^15^; Sébastien, Guigou, MD^2,16^; Hervé, Rouhette MD^2,17^; Emilio, Rapizzi, MD, PhD^18^; Eric, Denion, MD, PhD, FEBO^2,19^; Olivier Rebollo, MD^2,20^; Franck, Meyer, MD, PhD^2,15^; Joel, Uzzan, MD^21^; Marco, Mafrici, MD, PhD^22^; Daniela Bacherini, MD, PhD^23^; Stefania, Favilla, PhD^24^; Guido, Ricciotti, MD^8^; Salvatore, A, Tedesco, MD^8^; Stefano, Gandolfi, MD^8^; Marc, Muraine, MD, PhD.^1^

1. Ophthalmology Unit,”Centre Hospitalier Universitaire Charles-Nicolle”. Rouen. France.

2. P 1.5 Group. 80, allée des Ormes, 06250 Mougins. France.

8. Ophthalmology Unit, University Hospital of Parma, Parma, Italy.

9. Department of Basic Medical Sciences, Neurosciences and Sense Organs, University of Bari "Aldo Moro", Bari, Italy.

10. Department of Ophthalmology, San Marino State Hospital, Republic of San Marino.

11. Ophthalmology Unit, Ospedale Regionale Parini, Aosta, Italy.

12. Retina Unit, Ophthalmology Department,” Hôpital Pierre Paul Riquet”, CHU Toulouse, TOULOUSE Cedex 9, France; CERCO UMR 5549, CNRS-Université Toulouse 3, Toulouse, France.

13. Service d’ophtalmologie,”Centre Hospitalier intercommunal de Toulon” – La Seyne-sur-Mer, Toulon, France.

14. Department of Surgical and Biomedical Sciences, Section of Ophthalmology, University of Perugia, S. Maria della Misericordia Hospital. Perugia. Italy.

15. Department of Vitreoretinal Surgery, The Rotterdam Eye Hospital, Rotterdam, The Netherland.

16. AixVision. Aix-en-Provence. France.

17. Ophthalmology Center, « Hôpital Privé Arnault Tzanck Mougins Sophia-Antipolis». Mougins. France.

18. Ophthalmology Unit. “Dell’Angelo Hospital”. Mestre-Venice. Italy.

19. “Centre Ophtalmologique du Pays des Olonnes”. Les Sables-d'Olonne. France.

20. “Centre Rétine Sud”. Montpellier Métropole. France.

21. “Clinique Mathilde”. Rouen. France.

22. Ophthalmology Unit, “Centre Hôpital Alès-Cévennes”, Alès. France.

23. Eye Clinic, Neuromuscular and Sense Organs Department, AOU Careggi University Hospital,  Florence, Italy.

24. Independent Researcher in Biostatistics, on behalf of the Department of Medicine and Surgery of the University of Parma, Parma, Italy.
